# Machine learning-based prediction of stone-free rate after retrograde intrarenal surgery for lower pole renal stones

**DOI:** 10.1007/s00345-025-05762-7

**Published:** 2025-07-12

**Authors:** Hsiang Ying Lee, Yu-Hung Tung, Jose Carlo Elises, Yen-Chun Wang, Vineet Gauhar, Sung Yong Cho

**Affiliations:** 1 Department of Urology, Kaohsiung Medical University Hospital, Kaohsiung Medical University, Kaohsiung, Taiwan; 2 Department of Urology, School of Medicine, College of Medicine, Kaohsiung Medical University, Kaohsiung, Taiwan; 3 Graduate Institute of Clinical Medicine, College of Medicine, Kaohsiung Medical University, Kaohsiung, Taiwan; 4 Present Address: Department of Urology, Seoul National University College of Medicine, Seoul National University Hospital, Seoul, South Korea; 5 Ng Teng Fong General Hospital, Singapore, Singapore; 6 Asian Institute of Nephro-Urology, Hyderabad, India

**Keywords:** Machine learning, Lower pole renal stone, Stone free rate

## Abstract

**Background:**

Lower pole renal stones (LPS) present unique challenges for retrograde intrarenal surgery (RIRS) due to unfavorable anatomical features, often resulting in suboptimal stone-free rates (SFR). Recent advancements in machine learning (ML) offer new opportunities to predict surgical outcomes and guide clinical decision-making. This study aimed to develop and validate ML-based models to predict SFR following RIRS for LPS.

**Materials and methods:**

We retrospectively analyzed data from 327 patients with LPS who underwent RIRS at two academic institutions: Kaohsiung Medical University Hospital (KMUH, *n* = 193) and Seoul National University Hospital (SNUH, *n* = 134). Demographic, anatomical, and stone-related variables were collected, including stone burden, Hounsfield unit (HU), pelvic stone angle (PSA), and renal infundibular length (RIL). A Light Gradient Boosting Machine (LightGBM) algorithm was developed using KMUH data and externally validated with SNUH data. SHAP (SHapley Additive exPlanations) analysis was performed to interpret feature importance.

**Results:**

The LightGBM model achieved the highest predictive performance. External validation using the SNUH dataset yielded an accuracy of 77.1%, AUC of 0.759, and F1-score of 0.853. SHAP analysis revealed that stone burden, HU, PSA, and RIL were the most influential features. Notably, PSA demonstrated strong predictive relevance, supporting its use as an alternative to the traditional infundibulopelvic angle (IPA) in anatomical assessment.

**Conclusions:**

ML-based models, particularly LightGBM, offer robust predictive capability for SFR following RIRS in patients with LPS. These tools may enhance preoperative planning and personalized surgical strategies. Future prospective studies are warranted to further validate their clinical utility and expand on feature integration.

## Introduction

Lower pole stones (LPS) is the most common position of renal stones, and it comprised about 25–35% of renal stones [1]. LPS were considered as challenging position for retrograde intrarenal surgery (RIRS) due to the unique anatomical characteristics with difficulty in removing fragments. It can limit access for lithotripsy and make it difficult to extract stone fragments, even after comprehensive treatment [2]. Although laser technology, endoscopic instruments, and lithotripsy accessories have seen significant advancements in recent years, the stone-free rate (SFR) for LPS remains lower compared to stones in other locations [3].

The SFR for LPS treated with flexible ureteroscopy (fURS) remains suboptimal. One major factor is the presence of an acute infundibulopelvic angle (IPA) and a long, narrow infundibulum, both of which hinder access to the lower pole calyces [4, 5]). Although advancements in ureteroscope technology have improved deflection capabilities, the insertion of instruments such as laser fibers or stone retrieval baskets through the working channel can still significantly limit scope maneuverability [6]. To address these challenges and improve SFR, several techniques have been proposed. These include stone relocation from the lower pole to more accessible calyces [7], the use of smaller diameter laser fibers or baskets to enhance deflection, and the application of dusting modes that facilitate spontaneous clearance of stone fragments [8]. Additionally, the development and adoption of suction-assisted access sheaths have shown promise in fragment evacuation [9]. Despite these innovations, the implementation of targeted strategies specifically designed to optimize the management of LPS remains essential for improving surgical outcomes.

From previous literature, preoperative predictive scoring system or parameters were designed to predict the SFR of LPS. Stephanie L. Dresner et al. [2] demonstrated the role of IPA. The more acute IPA and larger preoperative stone size negatively affect SFR and need for repeat surgery. Yuleng Huang et al. [10] propose a scoring system comprised of stone characteristics, collecting system anatomy to predict the SFR of 1–2 cm LPS. Due to the difficulty of position and better decision-making before treatment plan, we conducted a machine-learning (ML) prediction model including different aspects of characteristics for better prediction [11].

## Methods and materials

We conducted a retrospective analysis of 327 patients with lower pole renal stones who underwent RIRS. Among them, 193 patients were treated at Kaohsiung Medical University Hospital (KMUH) between April 2018 and October 2023, and 134 patients were treated at Seoul National University Hospital (SNUH) from January 2022 to January 2024. Patients with rare or atypical conditions were excluded, as their limited numbers could have introduced bias and affected the generalizability of the results. Inclusion criteria were as follows: [1] age ≥ 18 years; [2] patients undergoing a single RIRS procedure without concurrent surgeries; [3] absence of specific conditions such as pregnancy, duplicated ureter, or horseshoe kidney; and [4] no prior ureteral stenting. This study was approved by the Institutional Review Board of KMUHIRB-F(I)-20,200,105 and SNUH1901-104-1005. Several patients and stone-related characteristics were used as input features for machine learning analysis, including age, sex, body mass index (BMI), stone laterality, number of stones, infundibular width (IW), pelvic stone angle (PSA) [12], average Hounsfield unit (HU) on computed tomography, stone burden, multifocality, and renal infundibular length (RIL).

All patients underwent preoperative computed tomography (CT) to evaluate relevant anatomical parameters, including pelvic stone angle (PSA), renal infundibular length (RIL), and infundibular width (IW). Stone burden was quantified by calculating the cumulative stone diameter (CSD). The average Hounsfield Unit (HU) of each stone was determined by measuring attenuation values at both the center and outermost edges of the stone on CT imaging. For patients with multiple stones, the mean HU was calculated as the average of all stones identified. Multifocality was defined as the presence of stones in more than one renal calyx. Stone-free status (SFS) was defined as the absence of visible stones or residual fragments ≤ 5 mm on follow-up CT scans obtained one month postoperatively. All imaging assessments were independently reviewed by two physicians.

### Model development, validation, and comparison

**Fig. 1:**
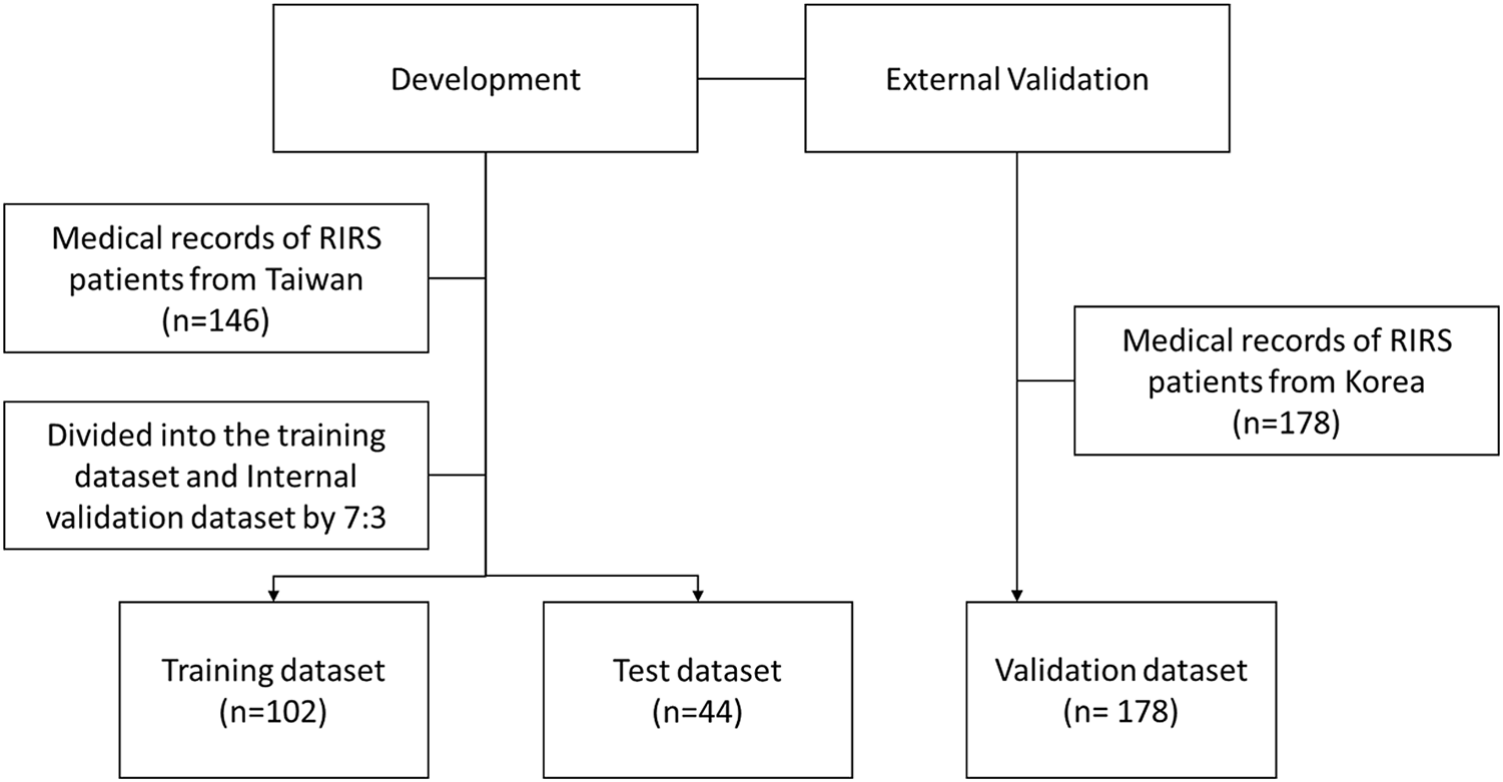
Flowchart of patient selection and dataset allocation for model development and validation

The prediction model based on ML was developed using the dataset from KMUH. It was preprocessing and training the model with the available algorithms using a 10-fold cross-validation. Hyperparameter tuning was automatically performed via random grid search. KMUH dataset was split into the training dataset (*n* = 135) for model development and test dataset (*n* = 58) for internal validation by 7:3. Then, the model would be external validated using the SNUH dataset (*n* = 134) (Fig. 1).

The accuracy (the proportion of all correct classification calculated by the formula: $$\:\:accuracy=$$$$ \frac{\:SF\:correct\:classification+non\:SF\:correct\:classification}{all\:classification}$$ ), AUC (area under the curve), Recall (sensitivity), Precision (Positive Predictive Value), F1-score (Harmonic mean of recall and precision:$$\:F1-score=\:\frac{2\times\:\:recall\times\:precision}{\:recall+precision}$$), Kappa (Cohen’s kappa coefficient), MCC (Matthews Correlation Coefficient) are the used to compare the accuracy of model according to different ML algorithms, and then the accuracy and F1-score is the principle indications to determine the best model for further exploration. The feature importance plot is used demonstrate the ranking of feature importance using gain importance.

SHAP (SHapley Additive exPlanations) is a game theory-based approach that can provide interpretability of ML models. It is another way to explain feature importance. The mean SHAP value of a feature indicates its contribution to the prediction. The positive or negative correlation between the feature and the predicted target depends on the mean SHAP value. In this study, features with the positive mean SHAP value mean that they have positive contribution to stone free (SF). On the *contrary*,* features with the negative mean SHAP value negative related to SF*,* also* can be considered positive related to non SF.

Individual SHAP values in a violin plot are used to interpret the correlation, red points present Individuals with higher feature value, and Individuals with lower feature value show as blue points. When the red points are located to the right of the central axis, it indicates a positive correlation between the feature value and SHAP. Conversely, if the blue points are distributed on the right side of the central axis, it signifies a negative correlation between the feature value and SHAP. The mean absolute SHAP values are ranked and shown in a bar chart to demonstrate the importance ranking of features. Python, along with the PyCaret and SHAP packages, are open-source and freely accessible.

### Statistical analysis

The associations between dependent and continuous variables were assessed using independent T-test (normal distribution) and Mann-Whitney U test (non-normal distribution), respectively. The assessment of association between the dependent and categorical variables was using Pearson’s chi-square test or Fisher’s exact test. Logistic regression was used to evaluate the relationship between SF and included features, and estimate the odds ratio.

Python (version 3.11; Python Software Foundation) is the principal software to perform the analysis, and the ML was implemented with the PyCaret 3.0.4 package. PyCaret is an open-source, low-code ML learning library, including 16 ML algorithms for classification (Appendix A).

## Results

The comparison of the training and test datasets is shown in Table 1. All of the characteristics are no significant difference between the training [Mean(Q1-Q3) age: 60(50–67) years; Men: 67.36%] and the test [Mean(Q1-Q3) age: 58 (48–67) years; Men: 65.67%] datasets.

**Table 1:** Comparison of patient characteristics between the training and test datasets

Characteristics	Training (*n* = 135)	Test (*n* = 58)	*P*
RIL, mm	24.2(4.6)	24.5(4.7)	0.6628
IW, cm	0.54(0.40–0.77)	0.55(0.41–0.75)	0.8771
PSA	45.7(11.6)	47.6(14.4)	0.3737
HU	712(473–980)	838(547–1150)	0.1470
BMI, kg/m^2^	26.7(24.1–29.1)	25.6(23.0-29.1)	0.2491
Age	60(50–67)	60.5(53–68)	0.2386
Stone number	2(1–2)	1(1–2)	0.5000
Stone burden, cm	1.65(0.95–2.54)	1.82(1.33–2.74)	0.2687
*Gender, n* (%)			0.9820
Women	44(32.60)	19(32.76)	
Men	91(67.40)	39(67.24)	
*Multifocal, n* (%)			0.3526
No	60(44.44)	30(51.72)	
Yes	75(55.56)	28(48.28)	
*Stone side, n* (%)			0.5781
Left	78(57.78)	31(53.44)	
Right	57(42.22)	27(46.56)	
*DM*			0.9642
No	81(60.00)	35(60.34)	
Yes	54(40.00)	23(39.66)	
*HTN*			0.4774
No	61(45.19)	23(39.66)	
Yes	74(54.81)	35(60.34)	

**Fig. 2:**
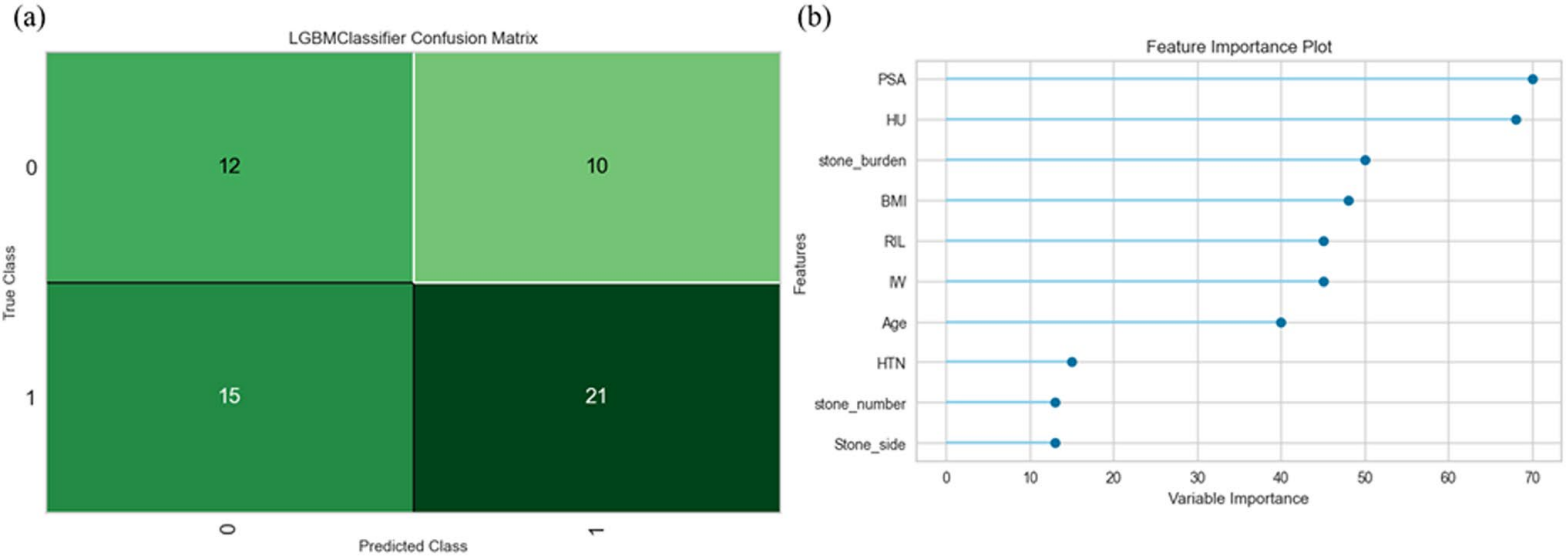
**a** Confusion matrix of the Light Gradient Boosting Machine (LightGBM) classifier applied to the internal test dataset. The matrix displays true positive [20], true negative [11], false positive [2], and false negative [14] counts, corresponding to the prediction of stone-free status [1] or non-stone-free status (0) after retrograde intrarenal surgery (RIRS). **b** Feature importance plot ranked by gain-based variable importance from the LightGBM model

The results of model training are shown in Table 2, LightGBM is the best classifier, with the highest accuracy in most indications except for Recall and AUC. In Fig. 2a, there is slightly difference of accuracy between the prediction for non-SF and SF, the confused matrix presents the accuracy in non-SF is 54.54% (12/22) and in SF is 58.33% (21/36). The total accuracy in the test dataset is 56.90% (33/58). According to gini importance, the main features order is: PSA, HU, stone burden, BMI, RIL, IW, and age (Fig. 2b).

**Table 2:** The model performance for different ML algorithms

Model	Accuracy	AUC	Recall	Prec.	F1	Kappa	MCC	TT
Light gradient boosting machine	0.7181	0.7153	0.8194	0.7594	0.7812	0.3712	0.3931	0.0790
Random forest classifier	0.6967	0.7438	0.7972	0.7444	0.7641	0.3276	0.3440	0.0260
Logistic regression	0.6731	0.7231	0.8083	0.7226	0.7514	0.2580	0.2900	0.0240
Naive bayes	0.6654	0.7158	0.8097	0.7087	0.7482	0.2414	0.2607	0.0100
Quadratic discriminant analysis	0.6511	0.6603	0.7625	0.7162	0.7312	0.2248	0.2450	0.0090
Ridge classifier	0.6429	0.7083	0.7847	0.6884	0.7241	0.1976	0.2145	0.0100
Decision tree classifier	0.639	0.609	0.7181	0.7240	0.7164	0.2105	0.2130	0.0100
Extra trees classifier	0.6374	0.7053	0.7764	0.6863	0.7252	0.1865	0.1990	0.0250
Linear discriminant analysis	0.6357	0.6992	0.7847	0.6833	0.7206	0.1739	0.1816	0.0100
Dummy classifier	0.6291	0.5000	1.0000	0.6291	0.7723	0.0000	0.0000	0.0100
Ada boost classifier	0.6214	0.6103	0.7500	0.6805	0.7048	0.1578	0.1782	0.0160
Gradient boosting classifier	0.6209	0.6947	0.7375	0.6822	0.7044	0.1643	0.1736	0.0190
SVM - linear kernel	0.6198	0.5994	0.6972	0.7535	0.6430	0.1681	0.2015	0.0090
K neighbors classifier	0.6082	0.6329	0.7417	0.6742	0.7042	0.1217	0.1185	0.0140

*AUC*, area under the curve; *F1*, F1-score; *κ*, Cohen’s kappa coefficient; *MCC*, Matthews Correlation Coefficient; *TT*, training time

The prediction model performance in different datasets is summarized (Table 3). The validation in the SNUH dataset demonstrates better accuracy (Accuracy: 0.7709, AUC: 0.7592, Recall: 0.8889, Precision: 0.8278, F1-score: 0.8534). For the all data (*n* = 327), the Accuracy is 0.6208, and the Recall and Precision are 0.7578 and 0.7026, respectively.

**Table 3:** Comparison of the accuracy in different datasets

Dataset	Accuracy	AUC	Recall	Precision	F1-score
Training (*n* = 135)	0.7181	0.7153	0.8194	0.7594	0.7812
Test (*n* = 58)	0.5690	0.5795	0.5833	0.6774	0.6269
KMUH (*n* = 193)	0.5745	0.5830	0.6853	0.6536	0.6657
SNUH(*n* = 134)	0.7709	0.7592	0.8889	0.8278	0.8534
ALL (*n* = 327)	0.6208	0.6096	0.7578	0.7026	0.7260

**Fig. 3:**
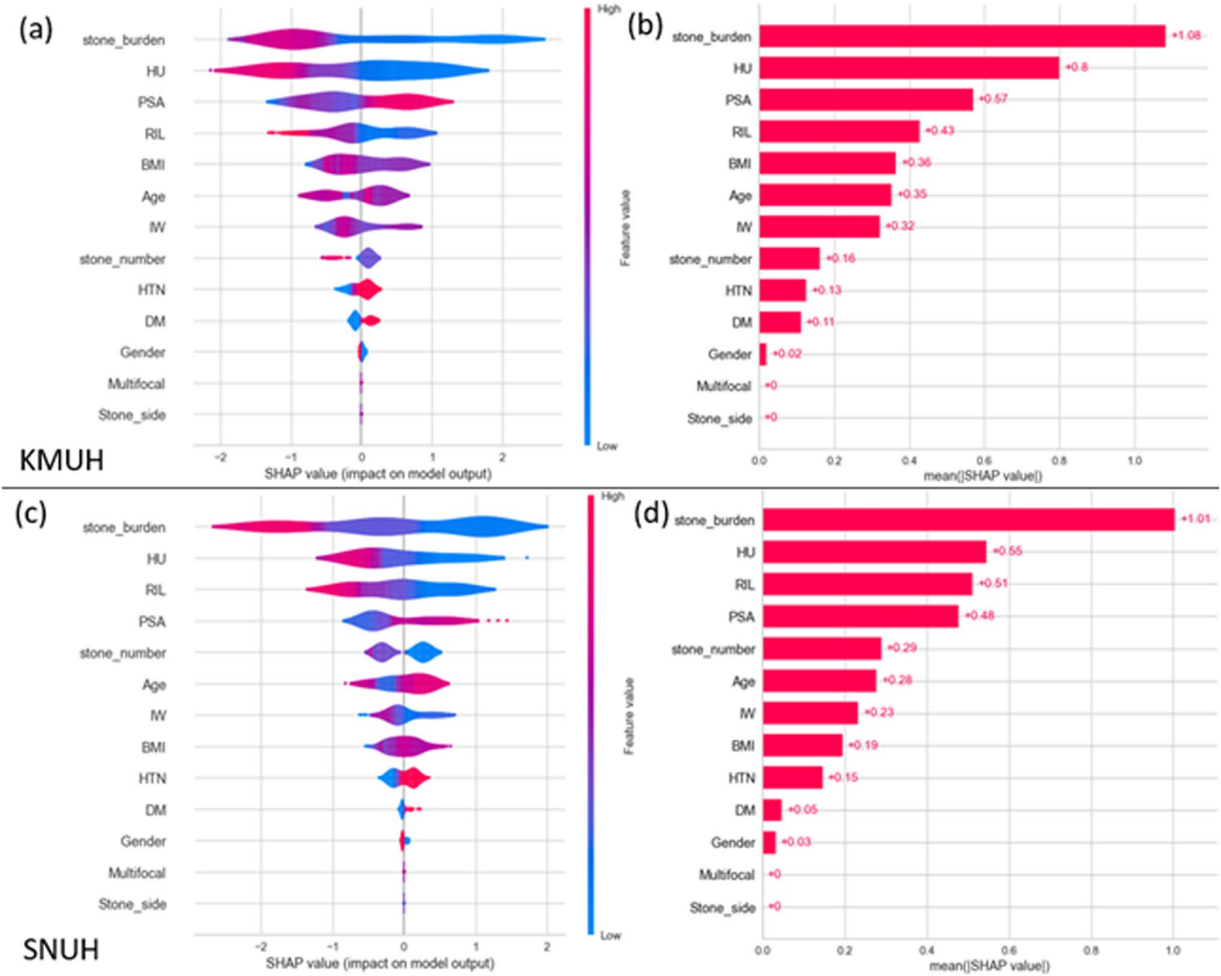
SHAP analysis of feature contributions to the prediction of stone-free status after retrograde intrarenal surgery (RIRS) in the LightGBM model. Violin plots (**a**, **c**) illustrate the distribution and impact of each feature’s SHAP value, with red indicating higher feature values and blue indicating lower values. Positive SHAP values are associated with higher predicted probability of stone-free outcome. Bar plots (**b**, **d**) rank features based on their mean absolute SHAP values, reflecting overall importance in model prediction

The SHAP analysis has highlighted the significance of features in the LightGBM model. As illustrated in Fig. 3a and b for KMUH and SNUH respectively, the violin plots indicate that higher values of stone burden, HU, RIL, and stone number correlate with a decreased prediction probability for SF. Conversely, individuals with elevated levels of PSA, age, DM, and HTN exhibit higher SHAP values. Those with either high or low BMI and IW demonstrate lower SHAP values compared to individuals with normal BMI and IW. The impact of gender, multifocality, and stone side on the model is minimal. These findings are consistent across both KMUH and SNUH.

Figure 3c and d present all features for KMUH and SNUH, respectively, arranged by their mean absolute SHAP values. The stone burden is the most significant contributor to SF, followed by HU. The factors influencing the prediction model between KMUH and SNUH differ, especially in BMI and stone numbers.

**Fig. 4:**
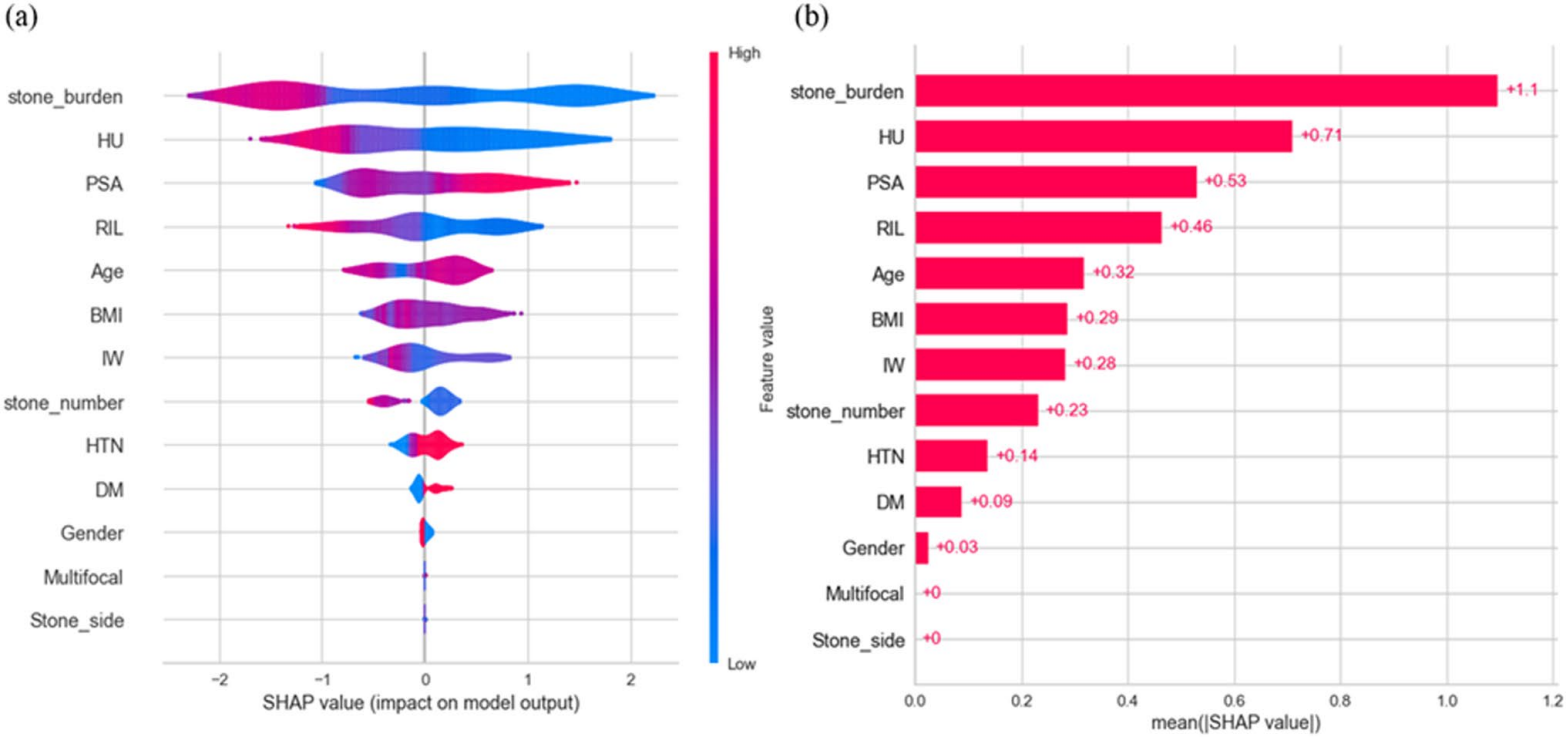
SHAP analysis of feature contribution in the combined dataset (KMUH + SNUH). **a** The violin plot, **b** The bar chart

The importance of stone and patient characteristics for all participants in this study is illustrated in Fig. 4, including both KMUH and SNUH. In addition to certain features with minimal contribution, such as DM, gender, multifocal, and stone side, there is a consistent relationship between features and SF in both logistic regression and machine learning analyses (see Table S2).

## Discussion

Based on the guidelines published by the American Urological Association (AUA) and the European Association of Urology (EAU), percutaneous nephrolithotomy (PCNL) is recommended as the first-line surgical intervention for renal stone larger than 2 cm [12]. In contrast, for renal stones measuring less than 2 cm, RIRS is generally considered the preferred approach. However, with ongoing advancements in fURS instruments and laser lithotripsy technologies, there has been a notable shift in clinical practice, with an increasing number of patients undergoing RIRS even for relatively large renal stones, instead of PCNL. RIRS has the advantages of fewer complications and faster postoperative recovery.

Even though, the treatment of LPS remains inherently more challenging than stones in other renal locations. Anatomical constraints such as a steep IPA, elongated and narrow infundibula, and limited calyceal mobility hinder access and effective fragment clearance [13]. Additionally, the presence of a laser fiber within the working channel compromises scope deflection, further complicating access to the lower pole. Giulioni C et al. large-scale study have demonstrated that larger stone burden, multiple stones, the use of reusable scopes are independently associated with residual fragments following RIRS [14]. These findings emphasize the necessity of individualized surgical planning for LPS, including detailed preoperative anatomical assessment and careful selection of surgical strategies and instruments to optimize clinical outcomes.

For accurate evaluation of the success of RIRS for LPS and prediction of SFR, several scoring systems were designed. The Resorlu-Unsal Stone Score (RUSS) scoring system incorporates several key parameters, including stone size, stone multiplicity, the presence of lower pole calculi with a renal IPA less than 45°, and abnormal renal anatomy such as horseshoe or pelvic kidneys [15]. Another widely used scoring system is R.I.R.S scoring system which was summarized by Xiao et al. in 2017 [16]. The system includes stone density, stone burden, renal infundibulopelvic length (RIL) and whether the stone is located in the lower calyx. As new technologies appear, the application of artificial intelligence (AI) including ML in endourology filed has dramatic development. From a previous research, it showed the increasing volume of related publications underscores a growing recognition of AI as a valuable tool in urolithiasis [17].

Various AI models have been integrated to enhance accuracy in tasks ranging from predicting spontaneous stone passage, predicting SFR, diagnostic prediction, imaging interpretation, stone composition analysis, procedural outcome prediction and treatment planning [18]. Kadlec et al. developed a predictive model aimed at prediction of SFR of various endourological procedures. It demonstrated a sensitivity of 75.3% and a specificity of 60.4% in predicting stone-free status, which was defined as the absence of stones on KUB X-ray or the presence of residual fragments less than 4 mm on CT. While the model exhibited high specificity (98.3%) in predicting the need for secondary interventions, its sensitivity in this regard was limited (30%). This investigation established a foundational framework for the future development of predictive nomograms in endourology [19].

Carlotta Nedbal et al. described if ML could predict RIRS outcome including stone free status after surgery. It identified total stone burden as the most influential positive predictor of failure to achieve stone-free status (SFS), followed by the presence of a preoperative ureteral stent. Conversely, factors inversely associated with SFS failure included a negative preoperative urine culture and stone location, particularly when confined to a single calyx or the ureter which has been confirmed that LPS indeed affect the SFR [20]. We demonstrate the potential of ML algorithms to predict SFR based on preoperative clinical parameters.

In order to identify the highest accuracy model, we both conduct internal and external validation. Among the ML algorithms, the Light Gradient Boosting Machine had the best SFR prediction. The SHAP values highlight the relative importance impact of each variable on the ML model’s prediction of SFR. In both cohorts, stone burden emerged as the most influential factor, exhibiting the highest mean SHAP values, indicating that it remains the most significant variable associated with postoperative SFR outcomes. Other important contributors included Hounsfield units (HU), PSA, and RIL, indicating the relevance of kidney anatomical parameters in predicting outcomes. On the contrary, patient demographics such as age and BMI demonstrated moderate influence, it had limited predictive weight in both models. Moreover, the consistent patterns across both institutions suggest model generalizability and external validity. These findings underscore the value of integrating comprehensive preoperative data, particularly stone-related characteristics into AI-driven prediction models to enhance individualized surgical planning and optimize patient outcomes in endourology. From this research, it also validates that the PSA angle measurement proposed by our team is indeed associated with postoperative SFR, suggesting that it could serve as a reliable alternative to the traditional IPA measurement [11].

Our study demonstrates the capability of ML algorithms to effectively handle complex and heterogeneous datasets, offering a significant advantage in predicting postoperative outcomes [21]. By incorporating patient demographic information and preoperative clinical variables, ML models are able to generate accurate and individualized prognostic estimates. However, there are some limitations. First, it is a retrospective research and needs to confirm in prospective trials. Second, we did not include all possible related parameters which can impact on postoperative SFR. Third, it was observed discrepancy in model performance across datasets, the accuracy on the test dataset may be limited because it has contained disproportionately challenging cases due to random data splitting. The study population should be increased to enhance the robustness of the model. Fourth, although stones smaller than 5 mm are generally considered passable on their own, many stone experts define true “stone-free” status as having residual fragments smaller than 2 mm visible on a postoperative CT scan at one month. Fifth, the external validation accuracy of 77.09% indicates the model’s potential applicability in clinical practice; however, additional prospective studies are warranted to confirm its generalizability before broader clinical adoption. However, this study confirms the feasibility of using ML algorithms as a tool for predicting postoperative outcomes. It also paves the way for future applications of ML in large scale data analysis, which may further enhance predictive accuracy over time. While ML holds great potential in healthcare, its integration into postoperative care must be approached with caution, including concerns over data privacy, limited model interpretability, and the risk of algorithmic bias. Addressing these issues is essential to ensure the ethical and effective application of ML technologies in clinical practice.

## Conclusions

This study demonstrates the feasibility and clinical utility of ML models in predicting SFR following RIRS for LPS. Among various algorithms, the Light Gradient Boosting Machine (LightGBM) achieved the highest predictive performance, with strong external validation in an independent cohort. The algorithms revealed that stone burden, Hounsfield unit, PSA which as a reliable alternative to the traditional IPA, and RIL were the most influential predictors of SFR, emphasizing the importance of both stone characteristics and renal anatomy in outcome prediction. These findings support the integration of ML-based decision support tools into preoperative planning for individualized surgical strategies. Future prospective studies with larger datasets and additional clinical variables are warranted to further refine and validate ML-based predictive models in endourology.

## Data Availability

No datasets were generated or analysed during the current study.
